# T59. VIRTUAL REALTY ASSESSMENT OF FUNCTIONAL CAPACITY IN EARLY SCHIZOPHRENIA: ASSOCIATIONS WITH NEUROCOGNITION, FUNCTIONAL CAPACITY PERFORMANCE, AND DAILY FUNCTIONING

**DOI:** 10.1093/schbul/sby016.335

**Published:** 2018-04-01

**Authors:** Joseph Ventura, Tamara Welikson, Kenneth L Subotnik, Arielle Ered, Richard Keefe, Gerhard H Hellemann, Keith H Nuechterlein

**Affiliations:** 1 UCLA Semel Institute for Neuroscience & Human Behavior; 2 Duke University

## Abstract

**Background:**

Research using virtual reality assessment of functional capacity has shown promise as a reliable and valid way to assess treatment response in patients with established schizophrenia. There has been little work on virtual reality based assessments of functional capacity for patients in the early phase of schizophrenia. We examined whether virtual reality based assessment methods reveal functional capacity deficits in young patients and relevant relationships with established measures of neurocognition, functional capacity performance, and daily functioning.

**Methods:**

The sample consisted of UCLA Aftercare Research Program patients (n=42) who were diagnosed by trained raters administering the SCID and who met criteria for schizophrenia, schizoaffective disorder, or schizophreniform disorder, and screened normal control subjects (n=13). Patients were within 2 years of their first psychotic episode upon clinic entry, were an average of 23.2 years old, and had an average of 12.9 years of education. The Virtual Reality Functional Capacity Assessment Tool (VRFCAT) was the computer-based measure of functional capacity. We used the MATRICS Consensus Cognitive Battery (MCCB) as an objective measure of neurocognition and the UCSD Performance-Based Skills Assessment (UPSA) to assess functional capacity performance. The Global Functioning Scale: Role and Social, and the Role Functioning Scale were used to assess work and school performance, familial interactions, and social functioning.

**Results:**

We were able to confirm that the deficit in functional capacity performance measured using VRFCAT is present in the early course of schizophrenia in that the patients were slower and committed more errors (M=830.41) as compared with normal controls (M=716.84; t=3.0, p<.01). Virtual reality based assessment of functional capacity was correlated with objective measures of neurocognition (MCCB Overall Composite), r=-.71, p=<.01, standard approaches to functional capacity assessment (UPSA), r=-.66, p=<.01, work and school functioning (r=-.52, p<.01), and level of social relationships (r=-.43, p=<.03), but not familial relationships (r=-.03, p=.87). Interestingly, neither neurocognition (MCCB) nor functional capacity performance (UPSA) were correlated with the level of familial relationships.

**Discussion:**

We extend previous findings in that even patients in the early course of schizophrenia showed virtual reality based functional capacity performance deficits when compared with normal control subjects. Virtual reality based performance was correlated with neurocognition, suggesting that it may be sensitive to changes in cognition. Furthermore, correlations with everyday work/school and social functioning indicate promise as a co-primary measure to index change in functioning in response to treatment. Interestingly, none of our measures of functional capacity or neurocognition were correlated with familial relationships indicating that the determinates of family interactions might be driven by factors other than cognitive capacities.

